# Consequences of exposure to sexual harassment among women working in hospitality workplaces in Bahir Dar City, Ethiopia: a structural equation model

**DOI:** 10.1186/s13690-023-01024-3

**Published:** 2023-01-18

**Authors:** Mulugeta Dile Worke, Zewdie Birhanu Koricha, Gurmesa Tura Debelew

**Affiliations:** 1 College of Medicine and Health Sciences, Debre Tabor University, Debre Tabor, Ethiopia; 2 Department of Population and Family Health, Faculty of Public Health, Jimma University, Jimma, Ethiopia; 3 Department of Health Behavior and Society, Faculty of Public Health, Jimma University, Jimma, Ethiopia

**Keywords:** Physical health, Reproductive health, Hospitality industry, Ethiopia

## Abstract

**Introduction:**

Sexual harassment is undoubtedly widespread, and many countries have enacted laws to punish and prevent it as insulting behavior. However, its impacts on the job, psyche, and physical health, especially reproductive health, are still severe and noticeable. Thus, this study aimed to examine the impacts of sexual harassment on the job, psychology, physical health, and reproductive health of women in the hospitality industry.

**Methods:**

Institution-based cross-sectional survey was conducted between October 1 and November 30, 2021. Data were collected among 689 women who experienced sexual harassment in the hospitality industry. In selecting the participants, two-stage cluster sampling techniques were used. The data collection was carried out in two complementary ways. The model of structural equations examines the relationship between the experience of sexual harassment and coping with consequences. The associations were confirmed via AMOS 23.

**Results:**

Sexual harassment positively predicted job outcomes and negatively predicted physical health. In contrast, coping with sexual harassment positively predicts health at work and in the body and is negatively associated with health in reproduction. Physical health fully mediated the link between sexual harassment (β = 0.017, t = 0.85, *p* = 0.022) and reproductive health outcomes and partially mediated (β = -0.021, t = -1.235, *p* = 0.017) between sexual harassment coping and physical health. The interaction between sexual harassment experiences and work experiences also strengthens the negative relationship between sexual harassment experiences and physical health.

**Conclusions:**

The impact of sexual harassment on women’s reproductive health was investigated in this study. It expands awareness of the effects of sexual harassment exposure, how to survive it, and how to establish effective preventative strategies, particularly in the hospitality industry. Effective prevention depends on preventing psychological and physical health, ultimately improving reproductive health. Thus, safe workplace initiatives and reproductive health care services are needed. Hospitality organizations should also devise a strategy for providing a supportive environment that can significantly improve women’s health.

**Supplementary Information:**

The online version contains supplementary material available at 10.1186/s13690-023-01024-3.

## Introduction

Sexual harassment (SH) is sexual violence and discrimination that comprises gender harassment, unwanted sexual attention, and sexual coercion [1]. It is a significant concern for academics, practitioners, regulators, and policymakers [2, 3]. Women are much more likely than men to become targets of SH [4, 5]. About 75% of women aged 18 years and above have experienced SH worldwide [6]. Workplace SH in hospitality industries was reported by 42% of women in the USA [7], 74.6% of women in 27 European countries [8], 89% of women in Australia [9], 50% of women in the Nordic Region [10], and 60% of women in Taiwan [11]. It is also a significant public health concern in Sub-Saharan African (SSA) hospitality industries such as Accra, Ghana (49.4%) [2, 12], Cameroon (98.8%) [13], Zimbabwe (78%) [14], and South Africa (14%) [15].

The widespread nature of SH makes it a precursor for several consequences and has continually been addressed as a significant occupational hazard [16]. Currently, these consequences are increasing, particularly among women working in low and middle-income countries [17–19]. Although SH’S magnitude, perception, and coping strategies differ by culture, its effects on the job, psychology, and health, including reproductive health, are consistent [20, 21]. Sexual harassment is also a critical social and human resource issue [22], particularly in the hospitality industry [23, 24]. It has been linked to suicidal behavior [25] and reduced mental health [26, 27]. It is also a workplace injustice that contributes to disparities in occupational health [28, 29].

However, studies have focused on organizational culture and what organizations can do to prevent SH and boost reporting [30–32]. Similarly, few studies have explored the actual SH situation or how SH’s immediate responses influence female employees [33] in hospitality industries. Other studies revealed that female employees in hospitality industries were also affected by quick reactions to SH events [33, 34], which made it impossible for them to describe their experiences as SH. Due to this, female employees would have felt forced to report the SH or confront the harasser [35]. Those who label and report SH experience-related psychological distress decreased job satisfaction, are perceived as less trustworthy and feminine [36], and scorn and retaliation from their employers [37, 38].

Furthermore, qualitative studies conducted in hospitality industries and other workplaces revealed that the consequences of SH are multidimensional [34, 35] and include job-related, psychology-related, and health-related consequences [36, 37]. The job-related outcomes include declines in job satisfaction; withdrawal from their organization (i.e., distancing themselves from work either physically or mentally without actually quitting, having thoughts or intentions of leaving their job, and leaving their job); declines in organizational commitment (i.e., feeling disillusioned or angry with the organization); increases in job stress; declines in productivity or performance [1]; and Stockholm syndrome (i.e., feeling entrapment)[38]. In addition, the greater the frequency, intensity, and duration of sexually harassing behaviors, the more women report symptoms of depression, stress, anxiety, and generally adverse psychological well-being [1, 27, 39, 40], and were associated with an excess risk of both suicide and suicide attempts [25]. Sexual harassment also contributes to subsequent psychological distress [26, 36].

Moreover, although there is doubt that it is similar in low-income countries, evidence in high-income countries indicates that SH increases financial stress by precipitating job change and significantly altering women’s career attainment [41]. However, most studies focused on job-related outcomes; others focused on psychological outcomes, few focused on health-related outcomes, and almost no studies on reproductive health-related outcomes. Consequently, including the #MeToo movement, organizations and individuals tried different interventions and behaviors to prevent or deny SH [42, 43]. The #MeToo movement raised the public’s awareness of SH that women experience within organizations, captured the possibility of SH’s ubiquity within daily workplace functions, and made it clear that people should not be silenced [35, 44]. However, individually, women think of their experiences as something other than harassment to avoid negative consequences [41, 42]. Studies also assert that feminist identity, appropriate dress, and avoiding provocative behavior of attracting more unwanted sexual attention could enable women to be more perceptive and less tolerant of SH [43, 45]. Others studied psychosocial workplace initiatives and found no association modification [27]. Further, it recently received substantial recognition for organizational policies, procedures, training programs, and the funneling of concerns expressed regarding inappropriate behaviors through complaint procedures [46].

Nevertheless, studies on SH’s impact on negative organizational performance in hospitality industries still indicate a high staff turnover rate, low profitability, and negative organizational image [24]. In addition, the SH was connected to a significant loss of productivity and a severe threat to customer service standards and profitability [37, 47]. It could also create a sexualized and risky image for the hospitality industry as a working environment [48] and deter potential workers who cannot tolerate these behaviors [49]. It was indicated that women who work in hospitality workplaces are vulnerable [50, 51]. Sexual harassment is associated with personal and occupational well-being, risk-taking behaviors, drug, and alcohol abuse, chronic diseases, and pain [21, 40, 52–55], and two studies showed an association between SH and menstrual disorders [56, 57]. However, most SH-related studies did not consider women’s reproductive health consequences. These are the current concerns of female employees, organizations, and governments in hospitality industries comprising hotels, bars, restaurants, fast-food establishments, cafeterias, and taverns, among other establishments. Though many studies have reported associations between SH and job, psychological, and health outcomes, few have simultaneously tested all the model components, including reproductive health outcomes.

**Fig. 1:**
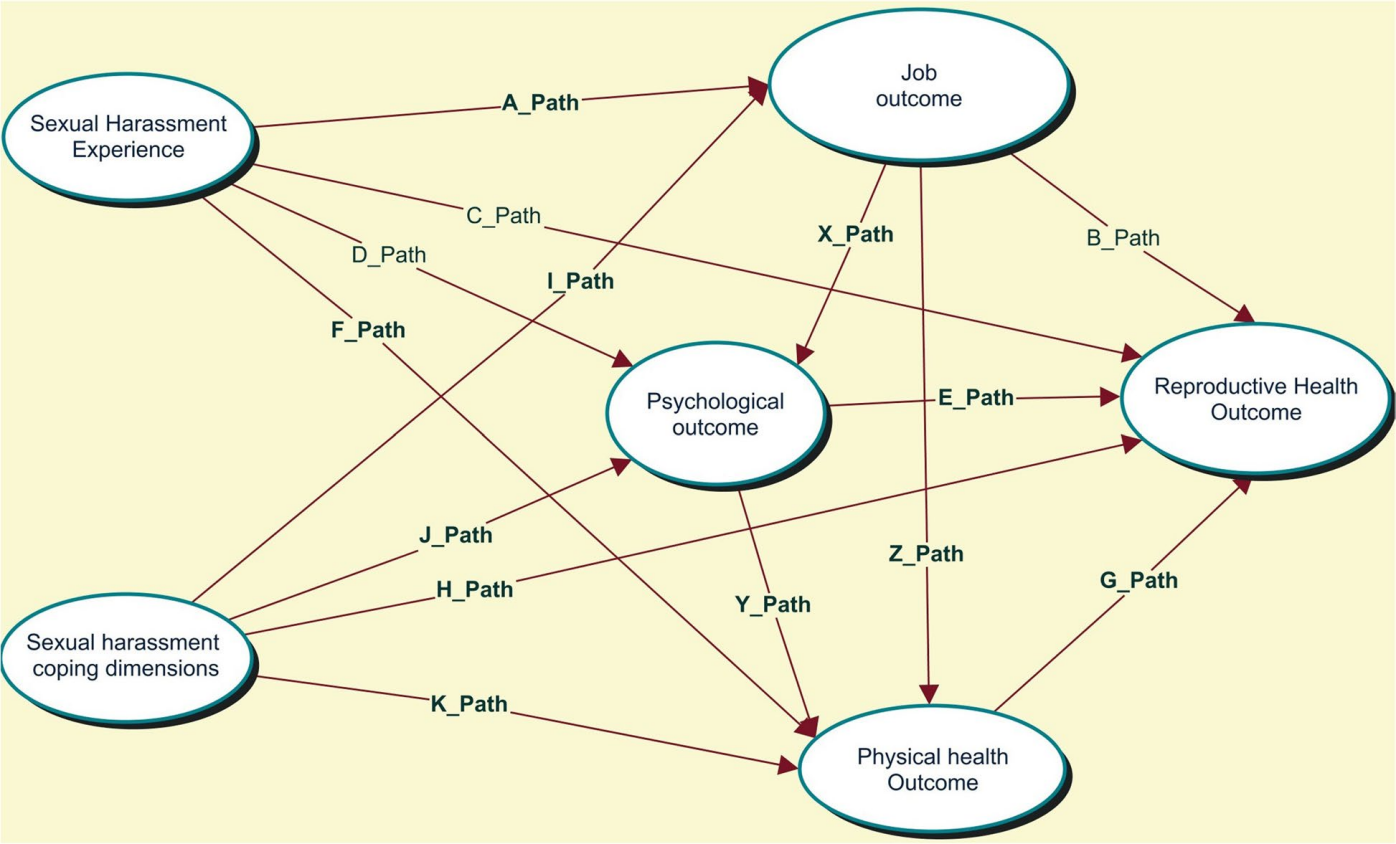
A planned model of SH, SH coping dimensions, job, psychological, physical health, and reproductive health consequences

The hospitality industry employs millions of women [58, 59] and provides numerous opportunities for empowerment and advancement, particularly in Ethiopia [60]. Its growth, size, diversity, and flexibility imply immense potential to empower and advance women economically, socially, and politically [61], and their work in this industry significantly improves their lives, families, and communities [62]. However, the effects of SH among women working in hospitality industries were not studied in Ethiopia. Therefore, the present study aimed to investigate workplace SH’s effect on job, psychology, and health, including reproductive health. Second, we aimed to examine how SH is linked to the consequences after controlling for other factors; and the direct, indirect, and total effects of exposure to workplace SH on outcomes, with a particular focus on reproductive health. Third, we investigated the mediating roles of job, psychological, physical health, and coping dimensions (Fig. 1).

### Hypothesis 1

Sexual Harassment coping techniques were expected to have an impact on women’s psychological health, physical health, organizational outcomes, and reproductive health outcomes as detrimental as sexual harassment experiences.

### Hypothesis 2

The relation between SH experiences and SH coping with women’s reproductive health outcomes will be mediated by psychological well-being and physical health, organizational outcomes, and directly associated with reproductive health.

### Hypothesis 3

The work experience of women hospitality employees moderates the relationship between SH experiences and physical heal health outcomes such that a higher level of work experience would strengthen the negative effect of SH on physical health outcomes.

These findings would have implications for the detailed understanding of consequences emerging from SH exposure, victims’ response to SH, and the development of effective prevention strategies, especially in hospitality workplaces. It would also help recommend related authorities to mitigate future occurrences of SH in the hospitality industry. Further, it is expected that this study’s findings would improve the planning of interventions to prevent both SH and its consequences**.**

## Methods

### Study setting

This study was conducted in Bahir Dar city, the third-largest city in the country, the business and political center of the Amhara National Regional State. Ethiopia is one of the destinations for tourists and accepts international rights to sexual and reproductive health. However, the status and factors of sexual and reproductive health rights were unknown in most tourist destination sites, including Bahir Dar city. Therefore, our study focused on SH in Ethiopia, particularly Bahir Dar. The study settings have been published elsewhere [57, 63, 64]. Bahir Dar is an ethnic, linguistic, and religiously diverse city that attracts all countries. It is a busy city whose inhabitants include people from all social classes. The existing masterplan of the Bahir Dar special zone includes four peri-urban cities (territories affected by robust expansion processes of the city, processes that are weakly opposed by marginal agricultural activities but where the expectations and interests of the communities are often high. Thus, they tend to have a chaotic and fragmented mix of urban and rural functions) and six urban sub-cities (*Kifle-Ketemas*) (are lower administrative units. They have their administrative heads, tax centers, land administration, and building permit offices, and are further divided into Kebeles, currently referred to as Woredas, that directly deal with the city’s inhabitants. The Kebeles or Woredas issue identification, maintain order and sanitation, control minor construction, and above all, own the bulk of the slum houses in the city). The peri-urban kebeles are Weramit, Addis Alem, Wereb, and Zenzelima, located immediately beyond the city boundaries, and the six urban sub-cities are Facilo, Gish Abay, Tana, Dagmawi Minilik, Belay Zeleke, and Atse Tewodros) [65]. Because the employment process was undertaken in the informal networks and the target groups are not beneficiaries of the formal social security schemes of the local government, the total number of women working in hospitality workplaces in the area is unknown. However, although there are no recent data on the number of hospitality workplaces and employees working in them, it has increased over the last decades.

### Study design and period

This institutional-based cross-sectional survey was conducted between October 1 and November 30, 2021.

### Population and sampling

The study population randomly selected women working in hospitality workplaces and who experienced sexual harassment. The survey’s source population was all women working in hospitality workplaces (hotels, restaurants, beverage groceries, and cafeterias) in the Bahir Dar city administration. Women who have been working in hospitality workplaces for at least six months, over 18 years, and in organizations with more than six employees were eligible for the study. Women who work in hospitality workplaces but do not directly contact customers, supervisors, and managers were excluded. To reduce selection bias and excessive inflation of the magnitude of SH, women who had been working in nightclubs and commercial sex workers were excluded.

An a-priori sample size calculator for structural equation models was used to calculate the sample size [52, 66, 67]. The sample size was calculated using the assumptions described below, which are required for a study that uses a structural equation model (SEM): the number of variables in the model (observed variables (150), latent variables (20)), the anticipated effect size = 0.3, the desired probability = 0.05 and statistical power levels = 80%. Accordingly, the calculator returned the minimum sample size required to detect the specified effect (227) and the minimum sample size required given the model’s structural complexity (538). Therefore, adding a 15% non-response rate, the recommended minimum sample size to detect the specified effect was 619. However, we found 689 women who had experienced SH in our prior study, and we included them all in this sample.

A multistage sampling technique was used to obtain the study population. First, the city was clustered into six subcities based on administrative classifications. A census was then conducted to get a list of hospitality workplaces and the number of female employees within each workplace in the six sub-cities. During the Census, the places of employment that provide hospitality services to the community were identified. All eligible hospitality organizations deemed functional for at least six months prior to the survey were identified. Different codes were provided to organizations and women, and the list of eligible organizations and the number of employees were identified and listed.

Then, three subcities (groups) (50%) were randomly selected (lottery method) [53]. Then, 50 organizations and 845 female employees from each facility were determined based on a probability proportional to size using the pool of female employees registered during the Census. Employees were systematically selected using the list during the Census. Finally, 689 employees who were identified as experiencing SH were all included in the consequences of the SH study (Supplementary Fig. 1).

### Measurements and operational definition

The exposure variables (exogenous constructs) were SH (any conduct perceived as unwelcome or undesired that women hospitality workplace employees view as offensive, creates an intimidating or offensive work environment that interferes with women’s ability to do their job) and SH coping (how people detect, appraise, deal with, and learn from stressful encounters) [54]. Sexual harassment experience was measured as mentioned above for the study of the consequences, but it was considered a Likert scale type. It was measured using 14-item Likert scale-type questionnaires, validated in a hospitality setting in Ethiopia [57]. The response options for the questions were 0 = never, 1 = once/twice, 2 = frequently, 3 = occasionally, and 4 = always. Similarly, SH coping was measured using 13-item Likert scale-type questionnaires, validated in hospitality settings in Ethiopia. The response options for the questions were 0 = never, 1 = once/twice, 2 = frequently, 3 = occasionally, and 4 = always. The dependent (endogenous construct) variable, reproductive health outcome, was a higher-order latent variable measured using transactional sex practice, premenstrual syndromes, and menstrual disorder questionnaires.

However, the mediating variables (exogenous and endogenous constructs) were the consequences for the organization, the psychology, and the physical health. Organizational and psychological variables were higher-order latent variables. So, they were measured by calculating the composites of each latent variable and considering them observable. Organizational outcomes were measured using job satisfaction, withdrawal, work withdrawal, organizational commitment, job stress, productivity or performance, and turnover intention. Each variable was latent but composed and considered the observable variable to measure the organizational outcome. The psychological outcome was also measured through subjective well-being, PTSD, mental health, and satisfaction with life. Each variable is latent but is composed of variables that can be observed to achieve psychological results. The covariates were socio-demographic characteristics, such as age, educational status, marital status, religion, ethnicity, employment, and monthly salary (Supplementary Table 1).

### Covariates

Socio-demographic variables include age, religion, ethnicity, educational level, work experience, income, distance from the institution, place of birth, marital status, living partner, personality, and employment status. A priori, possible covariates were established. They were age, gender, female-headed households, parental education, youth educational status, household income, place of residence, multiple risk factors (behaviors), and social support.

### Data collection methods

After the Census was conducted in the six sub-cities, an organization-based survey was conducted using an initially prepared questionnaire in English and translated into Amharic (the local language). It included various questions on socio-demographic characteristics, awareness, attitude, sexual harassment experiences, and organizational factors of sexual harassment in the workplace. It also included questions about sexual harassment experiences related to verbal, non-verbal, and physical harassment, the organizational contexts such as organization environment, workers’ power, workplace culture, gender composition, and organizational climate.

The enumerators, data collectors, and supervisors received a three-day intensive training to conduct the enumeration, identify the women, collect the data, and oversee to assure data quality. The training also included obtaining written consent (electronic), conducting face-to-face interviews, and navigating through the questionnaires in the Kobo toolbox platform preloaded into their smartphones, three training days followed by one day of fieldwork and one day of final tool talks. The study’s objectives and instruments were given by reviewing all the questions individually. Amendments and necessary modifications were made.

The selected hospitality industry owners/managers were approached and informed about the study, and their permission/collaboration sought to collect data from their sites. Once permission was granted, interviewers contacted the participants and their eligibility for participation.

The data collection was conducted in a quiet and private location. Data were collected during non-peak hours (morning and early afternoon) to enable women to participate fully. Women working in hospitality workplaces were usually busy during peak hours (late afternoon and evenings), promoting and serving customers. The questionnaire to collect the data was pre-tested for cross-cultural adaptability based on standard protocols [55, 68]. The data were collected by the Kobo Toolbox software [50] and a self-administered questionnaire (for sensitive topics, sexual harassment experiences, and transactional sex practices) using six trained female Bachelor of science holders in public health (two per sub-city). Besides, two Master of Public health holders from Bahir Dar University supervised the data collection process. Those professionals were recruited based on previous data collection experience (especially on sensitive topics) and fluency in the local language. Supervisors and the principal investigator supervised the data collection process, checked all the surveys daily, and provided immediate feedback to the data collectors as needed. The data sent to the Kobo toolbox server were checked daily for completeness, accuracy, and clarity. In the case of preliminary inquiries, the supervisors made the data collectors re-complete. We provided participants with tea, coffee, water, and soft drinks, and we covered transportation costs. Volunteers in the community involved in a non-governmental organization (Mahibre Hiwot) based in the city where the study participants live helped the researchers reach out to females and provided counseling center numbers for recalling potentially harmful scenarios.

### Data management and analysis

The data from the Kobo toolbox was downloaded into an excel spreadsheet and exported to SPSS V. 24 for cleaning and analysis and exported to the SPSS AMOS version 23 software package for analysis of associations. After the appropriate coding, the data were entered using the Epi Info version 7 software in a well-prepared data entry template for the self-administered data. After the entrance, the data were rechecked its correctness and screened for missing, outlier values and data entry errors using the frequency distribution of the variables and observation of the entered data. Actual and suspected errors were validated against the raw data, and corrections were made.

Descriptive statistics were computed to describe the socio-demographic characteristics of participants. Data were described using the mean, median, standard deviation (SD), frequency, percentage, and graphs. The magnitude of significant effects was interpreted using the following categorization: minor effects are rc < 0.29, medium effects are 0.30 < rc < 0.49, and significant effects are r_c_ > 0.50 [51]. Actual and suspected errors were validated against the raw data before the analysis, and any necessary corrections were made. The reliability of each item was tested for each exogenous and endogenous mediator and the endogenous outcome variable. Exploratory and confirmatory factor analyses were based on factor scoring, error terminology, factor loading parameters, and principal components. Only items with a factor loading greater than 0.4 were included in each construct, and cross-loaded items on more than two factors were omitted. Finally, confirmatory factor analysis was used to test the relationship between each construct in the measurement model. Before testing the hypothesized mediation model, examined factors for SH, SH coping, physical health, psychological health, reproductive health, and job outcomes. For each factor structure, items were deleted when they had low factor loadings (i.e., < 0.4) and problematic standardized residuals (i.e., surpassing the threshold of |2.5|).

Three phases were involved in the analysis. First, the correlation was used to assess the independent association of SH experiences and SH coping strategies with components of job outcomes, psychological outcomes, physical health outcomes, and reproductive health, as well as the association of each mediator with each other and the dependent variable (reproductive health) using correlation. The latent constructs of the mediators were then examined to see if the hypothesized mediators mediated the observed connections, which was done using mediation analysis with multiple mediators. Mediation analysis helps comprehend how exposure variables influence dependent variables [69]. Finally, the moderation effect of the interaction of work experience and SH experiences on physical health outcomes was assessed.

To assess the unadjusted relationships between the exposure variables (SH experience and SH coping), potentially mediating factors (job outcome, psychological outcome, and physical health outcome), and the outcome (reproductive health), we first built initial path models with mediating variables in AMOS using the Structural Equation Modelling (SEM). The estimand function command, 5000 bootstrap, and Bias-corrected percentile method built fully adjusted multivariate mediation models. Exposure variables (SH experience and SH coping), mediating factors, and reproductive health were modeled as continuous variables. Hence, the entire path model, i.e., paths linking exposure variables to mediators, paths linking mediators to the outcome, and paths linking exposure variables to the outcome, represented a linear model. In addition, serial mediation is concerned with finding the indirect effect across multiple constructs was also conducted. The indirect effect was calculated by taking the product of all the intervening relationships from the independent (SH experience and SH coping) to the dependent variable (Reproductive Health). Furthermore, since both work experience and sexual harassment experience are continuous variables, mean centering was done before the interaction to see the moderation effect.

The indexes for SEM analysis were assessed according to the following values: χ^2^ /SD of between 3 to 5 provides a good fit, root mean square of error approximation (RMSEA) values $\le$ 0.05 indicate excellent fit, and standardized root mean squared residual (SRMR) < 0.08, and Pclose > 0.05. The assumptions of the model were checked. It was generally accepted that the normed fit index (NFI) and comparative fit index (CFI) assesses of ≥ 0.90 show excellent fit.

## Results

### Socio-demographic characteristics

Of the 689 respondents, 670(97.2%) were Amhara ethnicity, 591(85.8%) were of orthodox religion, and 657 (95.4%) were born in urban residences. Five hundred twenty-nine (76.8%) were single, and 305(44.3%) had completed a high school education level. Four hundred one (58.2%) were between the ages of 25–29 years, with a mean age of 25.6 (± 2.5). Regarding wealth, 504(73.1%) had low-income families, and 360(52.2%) got less than or equal to 1000 ETB per month (Table 1).

**Table 1:** Socio-demographic characteristics of women who had experienced sexual harassment in Bahir Dar city hospitality workplaces, Ethiopia, October 1 to November 30, 2021 (*n* = 689)

Variable		Frequency (%)
Ethnicity	Amhara	670 (97.2)
	Others*	19(2.8)
Religion	Orthodox	591(85.8)
	Protestant	52(7.5)
	Catholic	27(3.9)
	Muslim	19(2.8)
Marital Status	Single	529 (76.8)
	Married	107 (15.5)
	Separated	28(4.1)
	Others**	25(3.6)
Birth Place	Urban	657(95.4)
	Rural	32(4.6)
Age (years)	20–24	241(35)
	25–29	401(58.2)
	≥ 30	47(6.8)
	25.6 (± 2.5)	
Educational status	Unable to read and write	25(3.6)
	Able to read and write only	27(3.9)
	Primary education	64(9.3)
	Secondary education	305(44.3)
	College diploma and above	268(38.9)
Employment	Full Time	662(96.1)
	Part-Time	27(3.9)
Monthly Salary	≤ 1000 ETB	360(52.2)
	1001 to 2000 ETB	319(46.3)
	≥ 2001 ETB	10(1.5)

Others**: widowed and divorced; Others*: Oromo and Agew

### Work environments and characteristics

**Fig. 2:**
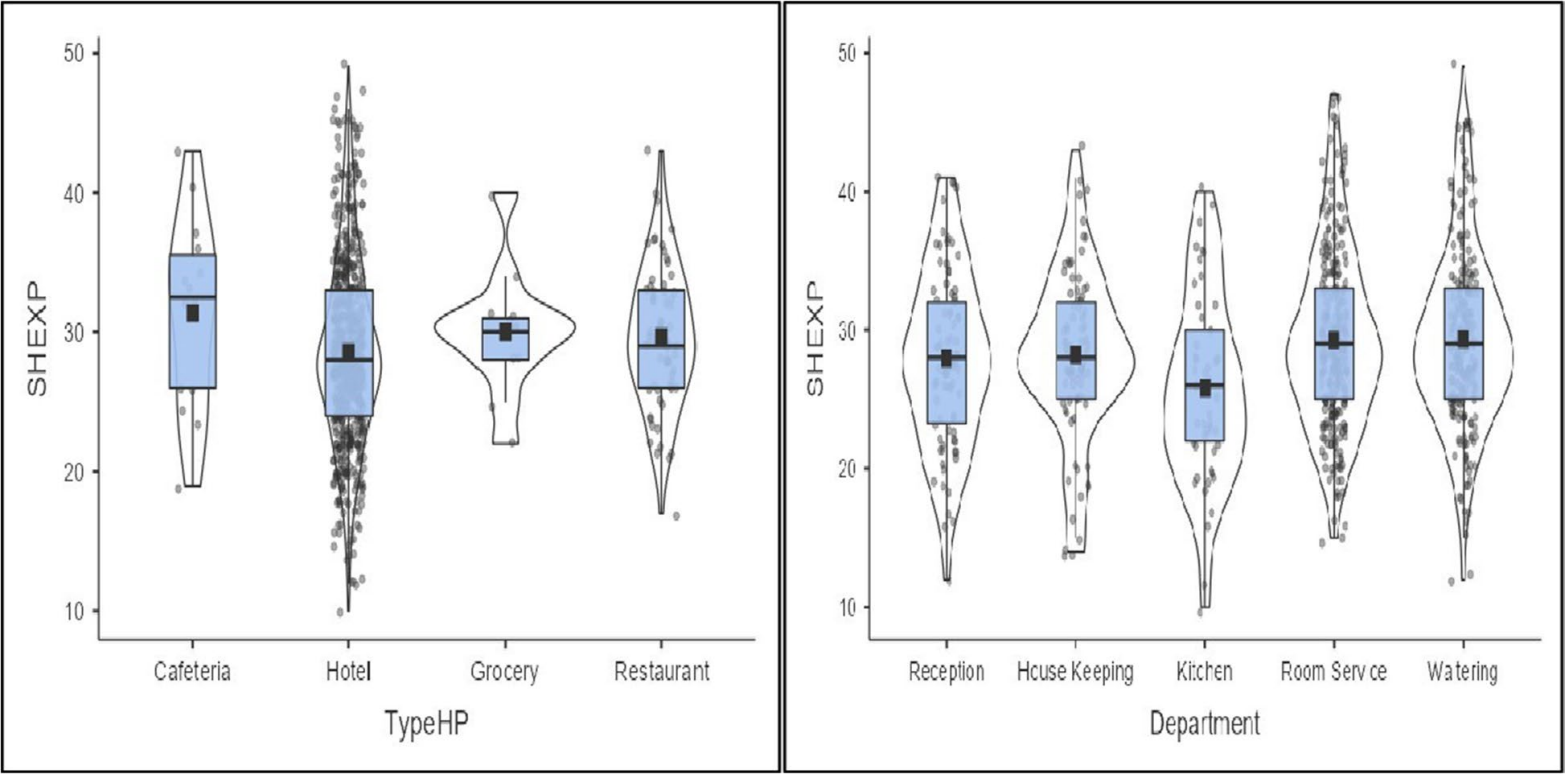
Distribution of women who experienced sexual harassment by workplace and departments in Bahir Dar city, Ethiopia, October 1 to November 30, 2021. *(TypeHP = type of hospitality workplaces, SHEXP = sexual harassment experience)*

Most women employees’ experienced SH in hotels and waiters’ departments (Fig. 2). Six hundred six (88%) of women are precarious employees. Employees work within a distance of 2.4 (± 4.3) kilometers and 8.3 (± 1.4) hours per day.

### Reproductive Health Outcomes

Regarding reproductive health outcomes, 361(52.4%) engaged in transactional sex in their lifetime, and 273 (39.6%) engaged in transactional sex within the previous 12 months. In addition, menstrual symptoms were experienced by all women, with a frequency being 160 (23.2%) experiencing rarely, 501 (72.7%) experiencing it occasionally, and 28 (4.1%) experiencing it frequently. Concerning menstrual disorder, 317 (46%) did miss menstrual periods after they experienced SH **(**Table 2**).**

**Table 2:** Experiences of menstrual disorder after Sexual Harassment Experiences among women working in Dahir Dar city hospitality workplaces, Ethiopia, October 1 to November 30, 2021 (*n* = 689)

Variable	Yes
Did you miss menstrual periods or variations in the length of time between periods of 7 days or more?	317(46%)
Did you experience the absence of a menstrual period in the last three months?	103 (14.9%)
Did you take hormone drug therapy for contraception or the treatment of menstrual irregularities in the previous year?	267(38.8%)
Have you been identified with polycystic ovary syndrome, ovary cysts, or uterine fibroids in the last year?	252 (36.6)

### Measurement model assessment

The measurement model was evaluated accordingly to confirm the construct’s reliability and validity [70]. Some items were removed when examining the measurement model because some factor loadings were below the suggested value of 0.400 [71]. Then, all loaded questions were computed into composite variables and involved in the final measurement process for organizational outcomes, reproductive health outcomes, and psychological outcomes. The Average Variance Extracted (AVE) and composite reliability of all the constructs equal or surpass the values of 0.50 and 0.70. Therefore, convergent validity and reliability were confirmed (Supplementary Table 2).

### Structural model assessment

The coefficient of determination (R^2^) results revealed the variance explained in the dependent variable because of the independent variable, R^2^ values are 0.55, 0.31, and 0.26 for psychological, organizational, and physical outcomes. The R^2^ values support the model’s in-sample predictive power [72] since it is above the required level of 0.10 [73]. Furthermore, effect sizes were computed to evaluate the number of exogenous variables contributing to the R^2^ value of an endogenous variable. The global model fitness also showed that the model is fit (χ^2^ = 1324, *p* < 0.001, CMIN/DF = 2.378, PClose = 1.00, CFI = 0*.*847, RMSEA = 0.045 (0.043, 0.047), SRMR = 0.086).

### The association of different outcomes with sexual harassment experiences & coping

The results indicated that menstrual symptoms (rc = 0.203; 95%CI [0.130, 0.274]) and transactional sex practice (rc = 0.192; 95%CI [0.119, 0.263]) had a significant positive association with SH experiences. Whereas women with premenstrual syndromes (rc = 0.401; 95% CI [0.337, 0.462]) had a stronger significant association, and transactional sex practice (rc = -0.126; 95%CI [-0.199, -0.052]) and menstrual disorders (rc = -0.2095% CI [(-0.27, -0.127]) had a significant negative association with SH coping techniques. While all psychological outcomes showed a stronger relationship with SH experiences, PTSS, depression, anxiety, and stress symptoms had a stronger relationship with SH coping mechanisms. Physical health is substantially correlated with SH coping mechanisms but not with SH experiences. Whereas all organizational outcomes were linked to SH Experiences, job satisfaction and organizational commitment were unrelated to SH coping strategies (Table 3).

**Table 3:** Results for the Correlates of women’s Sexual Harassment Experiences and coping techniques with organizational, psychological, physical, and reproductive health outcomes, Bahir Dar city, Ethiopia, October 1 to November 30, 2021

Variables	SH Experiences r_c_ (95%CI)	SH coping r_c_ (95%CI)
**Reproductive Health**		
Premenstrual syndromes	0.203(0.130, 0.274) ***	0.401(0.337, 0.462) ***
Menstrual Disorders	0.070(-0.004, 0.144)	-0.20(-0.27, -0.127) ***
Transactional sex practice	0.192(0.119, 0.263) ***	-0.126(-0.199, -0.052) ***
**Psychological Health**		
PTS disorder	0.133(0.059, 0.206) ***	0.076(0.001, 0.150) *
Subjective well-being	0.198(0.125, 0.269) ***	0.357(0.290, 0.421) ***
Current Health Satisfaction	0.269(0.198, 0.337) ***	0.092(0.017, 0.166) *
DAS symptoms	0.219(0.146, 0.289) ***	0.205(0.132, 0.276) ***
**Physical Health**		
Physical Health	-0.075(-0.148, 7.714e-5)	0.220(0.147, 0.289) ***
**Job Outcomes**		
Job Satisfaction	0.130(0.056, 0.202) ***	-0.013(-0.087, 0.062)
Job Performance	0.206(0.134, 0.277) ***	0.451(0.389, 0.509) ***
Job stress	-0.17(-0.242, -0.097) ***	-0.263(-0.331, -0.192) ***
Organizational deviance	0.20(0.127, 0.271) ***	0.332(0.264, 0.397) ***
Organizational commitment	0.215(0.143, 0.285) ***	-0.04(-0.114, 0.035)
Turnover intention	0.256(0.185, 0.324) ***	0.119(0.044, 0.192) *
Organizational withdrawal	0.212(0.139, 0.282) ***	0.106(0.031, 0.179) **
SH coping techniques	0.312(0.243, 0.378) ***	-

^*^p < 0.05, **p < 0.01, ***p < *0.001*

### The mediation analysis

The study’s results revealed that SH had a direct positive effect on job outcomes (β = 0.052, *p* = 0.048) and a negative effect on physical health outcomes (β = -0.704, *p* = 0.006). However, it did not affect psychological well-being (β = 0.043, *p* = 0.186) and reproductive health (β = 0.022, *p* = 0.313). On the other hand, SH coping had a positive effect on job outcomes (β = 0.081, *p* = 0.001), psychological outcomes (β = 0.041, *p* = 0.046), and physical health (β = 0.867, *p* < 0.001. However, it had a negative effect on reproductive health (β =—0.038, *p* = 0.038).

**Fig. 3:**
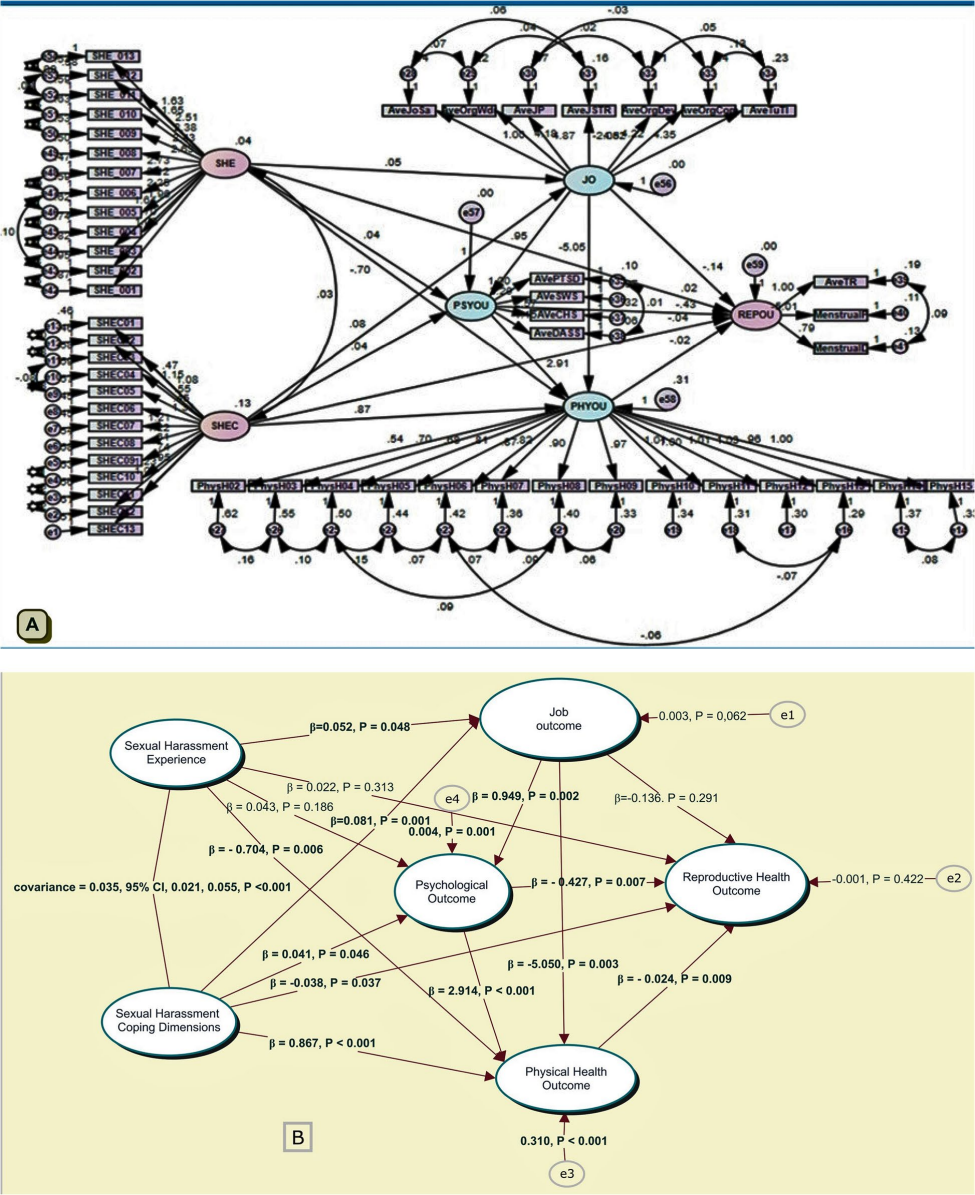
A fully mediated model with unstandardized regression weights, Bahir Dar city, Ethiopia, October 1 to November 30, 2021. *(SHE  = sexual harassment experiences, SHEC = sexual harassment experiences coping, JO =  Job outcomes, PSOU = psychological outcomes, PHYOU  = physical outcomes, REOU  = reproductive health outcomes)*

Similarly, the mediator variables were also assessed for their direct effect on reproductive health. Accordingly, job outcome had a positive effect on psychological outcomes (β = 0.949, *p* = 0.002), adverse effects on physical health (β = -5.050, *p* = 0.003), and did not affect reproductive health (β = 0.136, *p* = 0.291). On the other hand, the psychological outcome had a significant positive effect on physical health (β = 2.914, *p* < 0.001) and a negative effect on reproductive health (β = -0.427, *p* = 0.007). Lastly, physical health had a negative effect on reproductive health (β = 0.024, *p* = 0.009) (Fig. 3)**.**

The mediation analysis revealed that SH experiences had a direct (unmediated) effect on a physical health outcome (*p* = 0.015). However, SH experiences coping had a direct (unmediated) effect on the organizational outcome (*p* ≤ 0.001), physical health outcome (*p* = 0.001), and reproductive health outcome (*p* = 0.050). Therefore, SH had no direct effect on women working in hospitality workplaces’ organizational, reproductive health, and psychological outcomes. Similarly, SH experiences coping had no direct effect on psychological outcomes among women working in hospitality workplaces.

The effect of SH experience on reproductive health was fully mediated by physical health outcomes (β = 0.017, t = 0.85, *p* = 0.022), and the effect of SH experiences coping on reproductive health was partially mediated by physical health outcomes (β = -0.021, t = -1.235, *p* = 0.017). On the assessment of the serial mediating role of job outcome, psychological outcome, and physical outcome in different combinations on the relationship between SH experience and SH coping with reproductive health, the results revealed a significant indirect effect of SH coping on reproductive health through job outcome, psychological outcome and physical outcome (β = -0.005, t = -1, *p* = 0.009), job outcome and physical outcome (β = 0.010, t = 1.25, *p* = 0.011), and psychological outcome and physical health (β = -0.033, t = -0.75, *p* < 0.001). Furthermore, the direct effect of SH coping on reproductive health in the presence of the mediators was also significant (β =—0.038, *p* = 0.045). However, SH experiences had no serial indirect effect on reproductive health (Table 4).

**Table 4:** Summary of the mediation analysis of women’s Sexual Harassment Experiences and coping techniques with organizational, psychological, physical, and reproductive health outcomes, Bahir Dar city, Ethiopia, October 1 to November 30, 2021

Relationship	Direct effect	Indirect effect	CI		*P*-value	Conclusion
			LB	UB		
SH to RH	0.022 (0.298)					
SH to RH through JO		-0.007	-0.056	0.008	0.201	No mediation
SH to RH through PSYO		-0.018	-0.115	0.021	0.257	No mediation
SH to RH through PHYO		**0.017**	**0.001**	**0.080**	**0.022**	**Full mediation**
SH to JO-PSYO-PHYO to RH		-0.003	-0.022	0.000	0.057	No mediation
SH to JO to PSYO to RH		0.006	0.000	0.041	0.060	No mediation
SH to JO to PHYO to RH		-0.021	-0.104	0.000	0.053	No mediation
SHC to RH	**-0.038(0.045)**					
SHC to RH through JO		-0.011	-0.053	0.020	0.316	No mediation
SHC to RH through PSYO		-0.017	-0.065	0.006	0.144	No mediation
SHC to RH through PHYO		**-0.021**	**-0.064**	**-0.002**	**0.017**	**Partial mediation**
SHC to JO-PSYO-PHYO to RH		-0.005	-0.019	-0.001	**0.009**	**Partial mediation**
SHC to JO-PHYO to RH		0.010	0.001	0.032	**0.011**	**Partial mediation**
SH to PSYO-PHYO to RH		-0.033	-0.086	-0.009	** < 0.001**	**Partial mediation**

*SH* Sexual harassment, *RH* Reproductive Health, *JO* Job outcome, *PSYO* Psychological outcome, *PHYO* Physical health outcome, *SHC* Sexual harassment coping

### The Moderation analysis

**Fig. 4:**
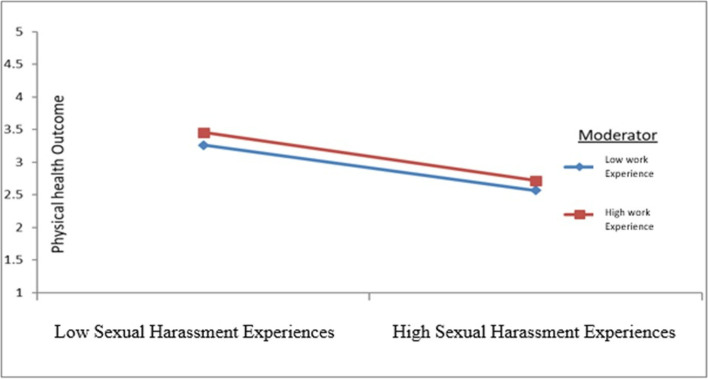
The moderating effect of women employees’ experiences in the relationship between sexual harassment experiences and physical health outcomes, Bahir Dar, Ethiopia, October 1 to November 30, 2021

Our hypothesis sought to ascertain the moderating effect of employee experiences between SH experiences and physical health outcomes. We tested for the interaction of experiences and SH experiences on physical health outcomes. Accordingly, the result revealed that the interaction between SH experiences and work experiences of women working in hospitality workplaces strengthens the negative relationship between SH experiences and physical health outcomes (β = -0.011, *p* < 0.001) (Fig. 4).

## Discussion

Sexual harassment and its coping mechanisms had correlations and different consequences, including consequences on reproductive health. Thus, the study anticipated examining the impact of SH experiences and SH coping on organizational, psychological, physical, and reproductive health outcomes and the indirect impact of SH experiences and SH coping on reproductive health outcomes in Bahir Dar city hospitality workplaces with the mediating effect of organizational, psychological and physical health outcomes. Additionally, it sought to examine the moderating effect of work experience between SH experiences and physical health outcomes.

First, consistent with a meta-analytic study [74], our research found that SH negatively affects all women’s job outcomes (job satisfaction, performance, stress, organizational deviance, commitment, turnover intention, and organizational withdrawal behaviors). Whereas, while women hospitality employees’ SH exposure increased, there was a decrease in physical health. This finding is consistent with a study conducted among the working women population in Quebec, Canada [75] and meta-analytic studies [74, 76]. However, this finding differs from an adolescent study [77]. Similarly, unlike other research [74], SH exposure was not directly predicted psychological [78] and reproductive health outcomes [79]. This finding clarified the indirect link between SH experiences and reproductive health outcomes such as menstrual disorder and menstrual symptoms. However, the mediation analysis with 2000 bootstrapping showed SH experiences had a direct (unmediated) effect on a physical health outcome, and physical health outcomes fully mediated the effect of SH experiences on reproductive health outcomes. This link indicates that the increment in physical health problems might also worsen reproductive health problems.

Second, job, psychological, and physical health outcomes increase while women cope with SH, worsening reproductive health outcomes. Sexual harassment experiences coping also had a direct (unmediated) effect on the organizational, physical, and reproductive health outcomes. Regarding the relationship between SH experience coping and the mentioned outcomes, our study agrees that coping with SH sometimes worsens job, psychological, and health outcomes [80, 81]. However, one of the essential findings in our study is the direct effect of sexual harassment coping on reproductive health outcomes, such as menstrual disorders, menstrual symptoms, and transactional sex practices. Physical health outcomes partially mediated the effect of SH experiences coping on reproductive health. The effect of SH experiences and SH experiences coping on reproductive health, on the other hand, is not mediated by psychological health or organizational results. The findings provide a solid theoretical contribution to the literature because there is limited research on the linkage of SH experiences and their coping mechanisms with reproductive health outcomes. This finding could also help us better understand the process of the outcome and mechanisms of action by researching the mediational process that clarifies how SH experiences coping affect reproductive health outcomes. However, we may uncover other, more effective intervention options [82].

Third, the interaction between SH experiences and the work experiences of women working in hospitality workplaces strengthens the negative relationship between SH experiences and physical health outcomes. This implies that prolonged exposure to SH worsens women’s physical health outcomes in hospitality workplaces. This, in turn, would lead women to experience reproductive health-related concerns such as menstrual disorders and menstrual symptoms. However, the mechanism by which physical and psychological health problems affect women’s reproductive health outcomes needs further research.

Finally, our study depicted that SH experiences coping predicted the above outcomes more than the SH experiences in hospitality workplaces in Bahir Dar city, Ethiopia. However, it is unlikely to mention the effect of coping without SH experiences because the SH experiences and the experiences of coping are significantly covaried. As a result, whether directly or indirectly, SH significantly impacted women’s reproductive health outcomes working in the hospitality industry, indicating that this study made a significant contribution. This study goes beyond the previous SH literature. First, unlike other studies which focused on its impact on service performance [89] and proactive customer service behavior [90], and workplace deviance [91], our study expanded outcome variables to reproductive health outcomes. Second, it is one of the first studies to examine the relationship between SH and the coping effects of SH on women in the hospitality industry. Third, investigating employment, psychological, and physical health outcomes as mediators adds to the body of knowledge by elucidating the mediating mechanism behind SH’s impacts on hospitality employees’ reproductive health outcomes. However, it is encouraged to replicate this study in other cultures and workplaces to validate the findings. Researchers may also consider a longitudinal study to show the link between SH and physical and reproductive health outcomes. Lastly, future studies should focus on other essential indicators (type of workplace, residence, perpetrators) to measure these factors’ impacts using a multilevel structural equation model.

These findings show that managers should take steps to prevent and mitigate the detrimental impacts of SH. First, managers must change their attitudes toward WSH, and businesses must strive to create an atmosphere of zero tolerance for WSH by building a sound policy system. Second, because enacting policies alone will not solve the WSH problem, the government and organizations should monitor their execution by advertising anti-SH policies. Organizations should also encourage highly experienced employees to seek professional aid or medical care and keep them from expressing dissatisfaction through damaging actions. There should also be a solid and well-supported complaint procedure in hospitality organizations. Governments should also safeguard employees against SH, particularly in developing countries. Additionally, governments should safeguard employees against SH, particularly in countries where the hotel industry is rapidly growing, and related regulation is lacking. Because the lack of a formal definition of SH announced by the central government makes it more challenging to identify and supervise SH [57, 83], governments should perfect the legal system to prevent WSH, including a formal and nationally consistent definition of WSH, clear punishment measures, and victim protection principles.

### Implications

Theoretically, this study’s findings contribute significantly to existing studies by demonstrating that SH coping predicted the outcomes mentioned earlier more than the SH experiences in hospitality workplaces. However, mentioning the effect of coping without SH experiences is unlikely because the SH experiences and the experiences of coping are significantly correlated. As a result, whether directly or indirectly, SH significantly affected the RH of women’s employees, propelling this study above and beyond previous SH literature. First, unlike previous studies that focused on its impact on service performance [83], proactive customer service behavior [84], and workplace deviance [85], this study broadened the outcome variables to RH outcomes. Second, it is one of the first studies to look at the relationship between SH and the coping effects of SH on women working in the hospitality industry. Third, investigating job, psychological, and physical health outcomes as mediators adds to the body of knowledge by elucidating the mediating mechanism behind SH’s effects on the RH of hospitality employees.

Practically, our findings are essential for developing effective strategies, norms, and policies to establish a healthy and respectful work environment in hospitality settings, thereby improving a variety of important individual, group, customer, and organizational outcomes. Thus, the themes acknowledged here signify current social dynamics in hospitality industries and women’s relationships with customers, co-workers, agents, and immediate bosses for policy and program implications. Organizations need to implement internal management protocols for reporting SH that serve as effective deterrents when such behavior is considered intolerable and complemented by appropriate actions to support victims without fear of retaliation. Secondly, it identifies an urgent need to improve management competence and supervision in dealing with difficult situations. It has also initiated the preference for a psychological and social risk assessment to detect such situations. Moreover, it signifies the requirement of a more outstanding commitment from the government and the participation of all parties involved.

The policy has been deafeningly silent on this alarming trend in hospitality workplaces. The findings of the study have generated a sense of awareness among policymakers. Because SH has significant consequences for decent work, prioritizing work decency in the policy agenda is crucial for achieving Goal 8 of the SDGs. Thus, the findings underlined stakeholder consultation requirements on acceptable customer behavior, as management discretion in dealing with unacceptable customer and manager behavior, which energizes workers to combat SH and increase their stake in the power relationship with customers. Likewise, it underlined the formulation and implementation of organizational SH policies and strategies in collaboration with governmental organizations (public tourism administrators, Ministry of education, Ministry of women and children, and general attorney), non-governmental organizations (local and international), and other relevant stakeholders like civic societies. In addition, it stated the importance of disseminating SH education materials, such as leaflets and hospitality posters, at public and private offices. The study also showed the necessity of assigning trained employees to handle SH complaints to ensure that victims are treated professionally without trivializing their complaints.

Owners and managers of hospitality workplaces should support their employees’ right to decent work and a safe working environment. Similarly, acceptable guest behavior should be written down and displayed prominently. Thus, by providing practical support and security to female employees and promoting gender-equitable attitudes among employees, customers, co-workers, & immediate bosses, the hospitality workplaces can be a stepping-stone rather than a barrier to females reaching their full potential. The researcher also suggests that hospitality workplaces collaborate with Ethiopian law and labor relations experts to improve SH policies that change deeply ingrained beliefs and norms and foster gender equality.

There are a few flaws in this research. First, we used a survey of women who had been sexually harassed during the previous six months. Although it reduces recall bias, the women were likely to forget their experiences. Similarly, using questionnaires with different response categories could cause range restrictions. As a result, future studies should use a longitudinal research design to detect recall bias. Second, this research was carried out in Ethiopia, where the culture differs from Western and Eastern countries, limiting the generalizability of our findings to high-income countries. Ethiopian hospitality personnel facing SH may be more humiliated to confide in others for comfort or to report to organizations for safety because of their goal to maintain interpersonal harmony and their conservative attitude regarding sex. As a result, the relationship between them has deteriorated. Finally, this study tried to see limited reproductive health outcomes, such as menstrual disorders, premenstrual symptoms, and transactional sex practices, which limit the generalization of the effect of SH on other reproductive health issues.

## Conclusions

The novel outcomes of current research suggest that SH and its coping predict job, physical, psychological, and reproductive health outcomes. It revealed that physical health fully mediated the link between sexual harassment and reproductive health outcomes and partially mediated between SH coping and physical health outcomes. The interaction between SH experiences and the work experiences of women working in hospitality workplaces strengthens the negative relationship between SH experiences and physical health outcomes. This study believes that effective prevention of SH depends on preventing psychological and physical health outcomes, ultimately improving reproductive health outcomes. The study findings also suggest establishing safe workplace initiatives and reproductive health care services for women working in hospitality workplaces. Therefore, hospitality workplaces should develop a strategy to deliver a supportive climate that can substantially increase women employees’ health, contour, and endure sexual harassment.

## Supplementary Information

**Additional file 1:** **Supplementary table 1.**

**Additional file 2:** **Supplementary table 2.**

**Additional file 3:** **Supplementary figure 1.**

## Data Availability

All data are kept in the manuscript.

## Abbreviations

AMOS: Analysis of Moment structure
AVE: Average Variance Extracted
CFI: Comparative Fit index
DASS: Depression, anxiety, and stress symptoms
ETB: Ethiopian Birr
IRB: Institutional Review Board
NFI: Normed fit index
PTSS: Post-traumatic stress symptoms
RMSEA: Root mean square error approximation
SEM: Structural equation Modelling
SH: Sexual Harassment
SRMR: Standardised root mean squared residual
SSA: Sub-Saharan Africa
