# Supplementary material for: Consequences of exposure to sexual harassment among women working in hospitality workplaces in Bahir Dar City, Ethiopia: a structural equation model
Source: Arch Public Health. 2023 Jan 18;81:7. doi: 10.1186/s13690-023-01024-3 (PMC9847057; doi:10.1186/s13690-023-01024-3)
Supplement: Supplementary file 1 — Additional file 1: Supplementary table 1. [file 13690_2023_1024_MOESM1_ESM.docx]

**Supplementary Table 1.** Operational definition and measurement of independent variables in the sexual harassment consequences study, Bahir Dar city, Ethiopia, October 1 to November 30, 2021.

| **Variables** | **Variable description and measurement** |
| --- | --- |
| **Job-related consequences** | |
| Job satisfaction | Positively affects employment and is a stable evaluation of how the job lights the employee’s needs, wants, or expectations. Job Satisfaction was measured using a questionnaire adapted from the Minnesota Satisfaction Questionnaire Short Scale, assessing participants’ job satisfaction after experiencing SH [1, 2]. It contains two subscales (intrinsic and external satisfaction) with 20 items. Items were divided into five levels, (1 = strongly unsatisfied, 2 = unsatisfied, 3 = uncertain, 4 = satisfied, and 5 = strongly satisfied). The higher the self-evaluation of the participants, the higher their satisfaction with the work. |
| Work withdrawal | It is defined as employees’ attempts to remove themselves from the immediate work situation while maintaining organizational membership. It comprised absenteeism, tardiness, and favoritism. A 13-item absenteeism scale measured it, consisting of a Likert scale questionnaire indicating a 5-point scale that ranges from 1 = sure to be absent and 5 = sure to go to work. The five-item tardiness scale was also measured, which required respondents to indicate the desirability, frequency, likelihood, and ease of engaging in these behaviors, and the nine-item unfavorable job behavior scale required respondents to show the rate with which they engaged in each act. |
| Organizational deviance | It was described as the deliberate (or intentional) desire to cause harm to an organization – more specifically, a workplace [3] and measured by a 12-item level of organizational deviance (deviant behaviors directly harmful to the organization) and a 7-item scale of interpersonal deviance (abnormal behaviors directly unhealthy to other individuals within the organization). |
| Turnover Intention | It is defined by employees’ intentions to leave their jobs and the organization itself [4]. It was measured by using a six-item turnover intention scale. All the items were measured using a five-point Likert scale ranging from strongly disagree (1) to agree (5) strongly. |
| Organizational commitment | It is the relative strength of an individual’s identification with and involvement in a particular organization. A self-administered questionnaire of 15 items captures the three factors [5] assessing “(1) the extent to which an individual identifies him/herself and involved in a particular organization; (2) a willingness to exert considerable effort on behalf of the organization; and (3) a strong desire to maintain membership in the organization. Six questions were negatively phrased and reverse coded, and five-point Likert scale response categories were used for all items. |
| Job stress | It is a condition that the individual employee frequently experiences due to increased work demands. 16-item Stress in General Scale is a global measure of general job stress using the job’s adjective checklist format [6]. On this scale, one indicates that the item produces no pressure, two produces little trouble, three creates some concern, four yields quite a bit of pain, and “5” provides great stress. It was done for both operational and organizational stress sources. |
| Productivity/performance | It is an essential element of organizational behavior research and has been considered a significant indicator of active organizations. The current study utilized the Job Performance Scale, which consists of 25 items, covering three extents of Job Performance (i.e., Altruism, Conscientiousness, and Task Performance) developed by Goodman SA and Svyantek DJ [7]. |
| **Psychological health-related consequences** | |
| Subjective Well-being Scale | Personal well-being concerns peoples’ self-reported assessment of their well-being - an individual’s appraisal of a person’s environmental circumstance, behavioral response, and the personal consequences of that process. It was measured by the revised BBC-SWB scale, which comprises 24 items in three underlying dimensions (psychological well-being, physical health and well-being, and relationship) [8]. Each scale allows multiple-choice answers with scores ranging from 1 (not at all) to 5 (extremely). All items, except one, were scored positively from 1 to 5, with 5 reflecting well-being. |
| Post-traumatic stress symptoms | Post-traumatic stress (PTSS) is the most common psychiatric sequelae associated with a traumatic injury. It was measured with a psychometrically valid 17-item self-report scale [9]. The answer options were 0 (not at all) to 3 (3 - 5 times per week/ very much/ always). |
| Mental health | DASS-21 was a validated and reliable psychological screening 21-item instrument to differentiate depression, anxiety, and stress symptoms. It was measured through depression, anxiety, and stress using Lovibond and Lovibond’s abridged version of the DASS-21 [10], validated in Ethiopia [11]. Participants were asked to indicate whether they had experienced symptoms in each field the previous week. The scoring was 0 (did not apply) to 3 (used most of the time). Scores from each dimension were summarised. The final score was multiplied by two and categorized according to the usual DASS manual: mild, moderate, severe, and too severe. Accordingly, a depression score of 0 - 9 was considered normal, 10 - 13 as mild, 14 - 20 as moderate, 21 - 27 as severe, and 28 and above as overly critical for depression. An anxiety score of 0–7 was considered normal, 8–9 was considered mild, 10–14 was considered moderate, 15–19 was considered severe, and 20 and above was considered overly critical. A stress score of 0–14 was considered normal for participants with stress, 15–18 as mild, 19–25 as moderate, 26–33 as severe, and 34 as overly critical. As a result, depression > 9, anxiety greater than 7, and stress > 15. |
| Satisfaction with Life Scale | Eight items with a 7-point Likert-type scale. Example item: ‘‘In most ways, my life is close to my ideal.’’ [12]. |
| **Physical health symptoms** | The presence and severity of health symptoms were measured using a shortened (15-item) version of Miller’s Abuse, Physical Symptoms, and Injury Survey [13]. Women were asked whether they had experienced 15 physical health symptoms in the previous two weeks and, if so, whether they had been not at all, a little, quite a bit, or immensely bothered or caused pain by each symptom. |
| **Reproductive health-related consequences** | |
| Menstrual Disorders | Deviation, discomfort, or pain differs from the regular monthly menstrual cycle. All menstrual disorders are premenstrual syndrome, heavy bleeding, painful menstruation, irregular periods, and amenorrhea. All five complications were examined as binary variables (0 = No, 1 = Yes). While one of the symptoms was present, the final menstrual disorder was considered. |
| Pre-menstrual syndrome | Pre-menstrual syndrome (PMS) is a cluster of cyclical physical and emotional symptoms that occur in the second half (luteal phase) of the menstrual cycle and stops with a start or after a few days of menses (follicular phase)[14]. It was measured by a diagnostic tool developed and validated by Algahtani and Jahrami (2014) [15]. This tool included 24 questions on PMS symptoms categorized into psychological (depressed mood, hopelessness, feeling guilty, anxiety/ worry, affective labiality, increased sensitivity toward others, feeling angry, easily irritated/ agitated, lack of interest, difficulty in concentrating, loss of control, and feeling overwhelmed); physical (lethargy/ fatigue/ decreased energy, increased appetite, craving certain foods, hypersomnia, insomnia, breast tenderness, breast engorgement or weight gain, headache, muscle, joint, abdominal, and back pain, and acne); and behavioral (symptoms interfering with relationships, work or school, or daily routine). Each question has a 5-point Likert scale (never, sometimes, often, always, and severely). |
| Transactional sex | It is characterized as non-commercial, non-marital sexual relationships based on the tacit expectation that sex was exchanged for financial support or other benefits [16-18]. It was assessed by asking women employees whether they had had sex/relationship with a man in the past 12 months to get things they needed, money, gifts, or other essential things [18]. The questions were two, which assessed their ever practice and their practice in the past 12 months, and the response options were 0 = No and 1= Yes”. |

References

1. Hirschfeld RR: **Does revising the intrinsic and extrinsic subscales of the Minnesota Satisfaction Questionnaire short form make a difference?** *Educational and Psychological Measurement* 2000, **60:**255-270.

2. Rannona MV: **The relationship between job insecurity, job satisfaction and organisational commitment in a mining organisation.** North-West University, 2003.

3. O'Leary-Kelly AM, Griffin RW: **Job satisfaction and organizational commitment.** *Psychology and policing* 2013**:**381-408.

4. Tnay E, Othman AEA, Siong HC, Lim SLO: **The influences of job satisfaction and organizational commitment on turnover intention.** *Procedia-Social and Behavioral Sciences* 2013, **97:**201-208.

5. Price JL: **Handbook of organizational measurement.** *International Journal of Manpower* 1997, **18:**305-558.

6. Queirós C, Passos F, Bártolo A, Faria S, Fonseca SM, Marques AJ, Silva CF, Pereira A: **Job stress, burnout and coping in police officers: relationships and psychometric properties of the organizational police stress questionnaire.** *International journal of environmental research and public health* 2020, **17:**6718.

7. Goodman SA, Svyantek DJ: **Person–organization fit and contextual performance: Do shared values matter.** *Journal of vocational behavior* 1999, **55:**254-275.

8. Pontin E, Schwannauer M, Tai S, Kinderman P: **A UK validation of a general measure of subjective well-being: the modified BBC subjective well-being scale (BBC-SWB).** *Health Qual Life Outcomes* 2013, **11:**150.

9. Rahman AF, Manatunga A, Guo Y, Peng L, Warnock M, Ressler KJ, Jovanovic T: **A latent class analysis of PTSD symptoms among inner city primary care patients.** *J Psychiatr Res* 2018, **98:**1-8.

10. Lovibond PF, Lovibond SH: **The structure of negative emotional states: Comparison of the Depression Anxiety Stress Scales (DASS) with the Beck Depression and Anxiety Inventories.** *Behaviour research and therapy* 1995, **33:**335-343.

11. Yeshaw Y, Mossie A: **Depression, anxiety, stress, and their associated factors among Jimma University staff, Jimma, Southwest Ethiopia, 2016: a cross-sectional study.** *Neuropsychiatric disease and treatment* 2017, **13:**2803.

12. Diener E, Emmons RA, Larsen RJ, Griffin S: **The Satisfaction With Life Scale.** *J Pers Assess* 1985, **49:**71-75.

13. Campbell J, Jones AS, Dienemann J, Kub J, Schollenberger J, O'Campo P, Gielen AC, Wynne C: **Intimate partner violence and physical health consequences.** *Arch Intern Med* 2002, **162:**1157-1163.

14. Raines K: **Diagnosing premenstrual syndrome.** *The Journal for Nurse Practitioners* 2010, **6:**224-225.

15. Algahtani HM, Jahrami HA: **The experience and severity of premenstrual syndrome among a Saudi sample using a newly developed Arabic language scale.** *Arab J Psychiatry* 2014, **5:**33-39.

16. Stoebenau K, Heise L, Wamoyi J, Bobrova N: **Revisiting the understanding of “transactional sex” in sub-Saharan Africa: a review and synthesis of the literature.** *Social Science & Medicine* 2016, **168:**186-197.

17. Wamoyi J, Stobeanau K, Bobrova N, Abramsky T, Watts C: **Transactional sex and risk for HIV infection in sub-Saharan Africa: a systematic review and meta-analysis.** *J Int AIDS Soc* 2016, **19:**20992.

18. Wamoyi J, Ranganathan M, Kyegombe N, Stoebenau K: **Improving the Measurement of Transactional Sex in Sub-Saharan Africa: A Critical Review.** *J Acquir Immune Defic Syndr* 2019, **80:**367-374.
