# Supplementary material for: Consequences of exposure to sexual harassment among women working in hospitality workplaces in Bahir Dar City, Ethiopia: a structural equation model
Source: Arch Public Health. 2023 Jan 18;81:7. doi: 10.1186/s13690-023-01024-3 (PMC9847057; doi:10.1186/s13690-023-01024-3)
Supplement: Supplementary file 2 — Additional file 2: Supplementary table 2. [file 13690_2023_1024_MOESM2_ESM.docx]

**Supplementary Table 2.** Reliability analysis of independent variables in the sexual harassment consequences study, Bahir Dar city administration, Ethiopia, October 1 to November 30, 2021.

1. Exposure variables measures reliability

1.1 SH Experiences

| Items | | Factor | | | Cronbach’s alpha if an item deleted |
| --- | --- | --- | --- | --- | --- |
| Code | Name | Verbal | Non-verbal | Physical |  |
| SEQ_HW10 | How often do perpetrators target you for rumors of sexual promiscuity? | .845 |  |  | .905 |
| SEQ_HW9 | How often does a perpetrator violate your boundaries? | .814 |  |  | .903 |
| SEQ_HW8 | How often does a perpetrator touch you in a way that makes you feel uncomfortable? | .771 |  |  | .904 |
| SEQ_HW5 | How often do perpetrators have unwanted sexual conversations with you? | .715 |  |  | .905 |
| SEQ_HW4 | How often does a perpetrator sexually assault you in public or in private? | .638 |  |  | .908 |
| SEQ_HW11 | How often do perpetrators insult you by targeting your sexual orientation? | .600 |  |  | .908 |
| SEQ_HW29 | How often do perpetrators make unwanted attempts to stroke, fondle, or kiss you? |  | .851 |  | .907 |
| SEQ_HW27 | How often do perpetrators make unwanted attempts to establish a romantic sexual relationship with you? |  | .757 |  | .904 |
| SEQ_HW26 | How often do perpetrators make you afraid that you would be handled by them poorly if you did not cooperate sexually? |  | .737 |  | .902 |
| SEQ_HW24 | How often do perpetrators make you feel you were being bribed with some reward to engage in sexual behavior? |  | .688 |  | .905 |
| SEQ_HW23 | How often do perpetrators gaze, leer, or ogle at you in a way that makes you feel uncomfortable? |  | .670 |  | .905 |
| SEQ_HW17 | How often do perpetrators unnecessarily expose themselves in front of you? |  |  | 0.97 | .911 |
| SEQ_HW16 | How often does a perpetrator make sexual assaults, attempts of rape, or actual rape? |  |  | .474 | .910 |
| SEQ_HW18 | How often do perpetrators threaten you by filing a complaint about your service to your supervisor because you refused a sexual request? |  |  | .404 | .906 |
| Cronbach’s alpha coefficient | | **.879** | **.872** | **.769** | **.912** |

- 1. SH coping

| Items | | Factor | | | Cronbach’s alpha if an item deleted |
| --- | --- | --- | --- | --- | --- |
| Code | Name | Normalization | Engagement | Help-seeking |  |
| SHCQ_HW25 | How often did you keep silent to respond to sexual harassment? | .893 |  |  | .859 |
| SHCQ_HW24 | How often did you tolerate sexual harassment? | .740 |  |  | .857 |
| SHCQ_HW23 | How often did you confront the perpetrator? | .696 |  |  | .860 |
| SHCQ_HW26 | How often did you ignore sexual harassment? | .674 |  |  | .862 |
| SHCQ_HW22 | How often did you reject the request for sexual harassment? | .590 |  |  | .866 |
| SHCQ_HW19 | How often did you consult a psychologist because of the sexual harassment? |  | .921 |  | .861 |
| SHCQ_HW20 | How often did you negotiate with the perpetrator? |  | .773 |  | .861 |
| SHCQ_HW18 | How often did you consult a health care provider because of sexual harassment? |  | .765 |  | .863 |
| SHCQ_HW21 | How often did you discriminate against the perpetrators? |  | .495 |  | .865 |
| SHCQ_HW9 | How often did you get sympathy and understanding from friends who have had the same problem? |  |  | .739 | .861 |
| SHCQ_HW11 | How often did you seek reassurance from those who know you best? |  |  | .736 | .867 |
| SHCQ_HW10 | How often did you talk to people about the situation because it makes you feel better? |  |  | .686 | .863 |
| SHCQ_HW8 | How often did you go to a friend for advice on how to change sexual harassment? |  |  | .659 | .863 |
| Cronbach’s alpha coefficient | | **.846** | **.835** | **.811** | **.871** |

2. Mediators

2.1 Reliability of Orgnizational outcome measures

| Latent Variable | Constructs | Item-code | Factor Loading | **Cronbach’s α** |
| --- | --- | --- | --- | --- |
| 1. Organizatiinal commitment | Factor 1 | ORGCOM#09 | 0.861 | 0.83 |
|  |  | ORGCOM#08 | 0.624 |  |
|  |  | ORGCOM#10 | 0.619 |  |
|  |  | ORGCOM#12 | 0.568 |  |
|  |  | ORGCOM#11 | 0.553 |  |
|  |  | ORGCOM#14 | 0.497 |  |
|  |  | ORGCOM#15 | 0.488 |  |
|  |  | ORGCOM#13 | 0.389 |  |
|  | Factor 2 | ORGCOM#06 | 0.847 | 0.71 |
|  |  | ORGCOM#05 | 0.480 |  |
|  |  | ORGCOM#07 | 0.431 |  |
| 1. Turnover Intention | Factor 1 | TI#02 | 0.875 | 0.73 |
|  |  | TI#01 | 0.567 |  |
|  |  | TI#03 | 0.489 |  |
|  |  | TI#06 | 0.929 |  |
|  |  | TI#05 | 0.368 |  |
| 1. Organizational Deviance | Factor 1 | Deviance#03 | 0.765 | 0.70 |
|  |  | Deviance#02 | 0.585 |  |
|  |  | Deviance#09 | 0.546 |  |
|  |  | Deviance#18 | 0.454 |  |
|  |  | Deviance#17 | 0.421 |  |
|  |  | Deviance#04 | 0.320 |  |
|  | Factor 2 | Deviance#06 | 0.632 | 0.72 |
|  |  | Deviance#11 | 0.555 |  |
|  |  | Deviance#05 | 0.494 |  |
|  |  | Deviance#07 | 0.473 |  |
|  |  | Deviance#10 | 0.393 |  |
|  |  | Deviance#16 | 0.381 |  |
|  |  | Deviance#01 | 0.345 |  |
|  |  | Deviance#19 | 0.319 |  |
| 1. Job performance | Factor 1 | JP#02 | 0.505 | 0.70 |
|  |  | JP#08 | 0.502 |  |
|  |  | JP#16 | 0.471 |  |
|  |  | JP#03 | 0.437 |  |
|  |  | JP#04 | 0.390 |  |
|  |  | JP#21 | 0.347 |  |
|  | Factor 2 | JP#23 | 0.502 | 0.72 |
|  |  | JP#10 | 0.463 |  |
|  |  | JP#06 | 0.430 |  |
|  |  | JP#20 | 0.347 |  |
|  | Factor 3 | JP#09 | 0.616 | 0.73 |
|  |  | JP#18 | 0.398 |  |
|  |  | JP#15 | 0.331 |  |
| 1. Organizational withdrawal | Factor 1 | ORGWD#27 | 0.564 | 0.74 |
|  |  | ORGWD#32 | 0.497 |  |
|  |  | ORGWD#34 | 0.491 |  |
|  |  | ORGWD#33 | 0.485 |  |
|  |  | ORGWD#26 | 0.473 |  |
|  |  | ORGWD#28 | 0.454 |  |
|  |  | ORGWD#31 | 0.451 |  |
|  |  | ORGWD#30 | 0.424 |  |
|  |  | ORGWD#25 | 0.377 |  |
|  |  | ORGWD#24 | 0.340 |  |
|  |  | ORGWD#35 | 0.337 |  |
|  |  | ORGWD#18 | 0.323 |  |
|  | Factor 2 | ORGWD#04 | 0.521 | 0.77 |
|  |  | ORGWD#08 | 0.445 |  |
|  |  | ORGWD#05 | 0.427 |  |
|  |  | ORGWD#07 | 0.420 |  |
|  |  | ORGWD#09 | 0.394 |  |
|  |  | ORGWD#06 | 0.382 |  |
|  |  | ORGWD#16 | 0.336 |  |
|  |  | ORGWD#17 | 0.325 |  |
|  |  | ORGWD#10 | 0.321 |  |
|  |  | ORGWD#03 | 0.302 |  |
|  | Factor 3 | ORGWD#01 | 0.609 | 0.74 |
|  |  | ORGWD#02 | 0.501 |  |
| 1. Job satisfaction | Factor 1 | JOSA#06 | 0.558 | 0.79 |
|  |  | JOSA#07 | 0.519 |  |
|  |  | JOSA#13 | 0.475 |  |
|  |  | JOSA#12 | 0.467 |  |
|  |  | JOSA#18 | 0.458 |  |
|  |  | JOSA#17 | 0.436 |  |
|  |  | JOSA#11 | 0.406 |  |
|  |  | JOSA#09 | 0.402 |  |
|  |  | JOSA#05 | 0.385 |  |
|  |  | JOSA#20 | 0.382 |  |
|  |  | JOSA#19 | 0.376 |  |
|  |  | JOSA#04 | 0.375 |  |
|  |  | JOSA#08 | 0.357 |  |
|  |  | JOSA#15 | 0.334 |  |
|  |  | JOSA#14 | 0.325 |  |
|  |  | JOSA#16 | 0.315 |  |
|  |  | JOSA#10 | 0.313 |  |
|  | Factor 2 | JOSA#02 | 0.708 | 0.74 |
|  |  | JOSA#01 | 0.575 |  |
| 1. Job Stress | Factor 1 | JOSTR#01 | 0.759 | 0.76 |
|  |  | JOSTR#02 | 0.722 |  |
|  |  | JOSTR#11 | 0.566 |  |
|  |  | JOSTR#10 | 0.534 |  |
|  |  | JOSTR#03 | 0.479 |  |
|  |  | JOSTR#12 | 0.387 |  |
|  |  | JOSTR#09 | 0.340 |  |
|  |  | JOSTR#04 | 0.316 |  |
|  | Factor 2 | JOSTR#06 | 0.640 | 0.70 |
|  |  | JOSTR#14 | 0.501 |  |
|  |  | JOSTR#05 | 0.496 |  |
|  |  | JOSTR#15 | 0.443 |  |
|  |  | JOSTR#13 | 0.425 |  |
|  |  | JOSTR#08 | 0.389 |  |
|  |  | JOSTR#07 | 0.375 |  |
|  |  | JOSTR#16 | 0.301 |  |

2.2 Physical outcome measure reliability analysis

| Latent Variable | Constructs | Item-code | Factor Loading | **Cronbach’s α** |
| --- | --- | --- | --- | --- |
| Physical health | Factor 1 | PhysH#12 | 0.750 | **0.92** |
|  |  | PhysH#09 | 0.745 |  |
|  |  | PhysH#13 | 0.742 |  |
|  |  | PhysH#10 | 0.738 |  |
|  |  | PhysH#11 | 0.737 |  |
|  |  | PhysH#15 | 0.736 |  |
|  |  | PhysH#08 | 0.708 |  |
|  |  | PhysH#14 | 0.702 |  |
|  |  | PhysH#07 | 0.698 |  |
|  |  | PhysH#06 | 0.670 |  |
|  |  | PhysH#05 | 0.663 |  |
|  |  | PhysH#04 | 0.588 |  |
|  |  | PhysH#03 | 0.556 |  |
|  |  | PhysH#02 | 0.442 |  |

2.3 Psychological outcome measures

| Latent Variable | Constructs | Item-code | Factor Loading | **Cronbach’s α** |
| --- | --- | --- | --- | --- |
| 1. Depression, anxiety, and stress | Factor 1 | 0.383 | DASS#16 | **0.70** |
|  |  | 0.380 | DASS#09 |  |
|  |  | 0.376 | DASS#21 |  |
|  |  | 0.373 | DASS#14 |  |
|  |  | 0.368 | DASS#07 |  |
|  |  | 0.364 | DASS#10 |  |
|  |  | 0.363 | DASS#05 |  |
|  |  | 0.363 | DASS#19 |  |
|  |  | 0.349 | DASS#13 |  |
|  |  | 0.346 | DASS#06 |  |
|  |  | 0.340 | DASS#02 |  |
|  |  | 0.340 | DASS#17 |  |
|  |  | 0.337 | DASS#20 |  |
|  |  | 0.331 | DASS#03 |  |
|  |  | 0.321 | DASS#18 |  |
|  |  | 0.311 | DASS#11 |  |
|  |  | 0.303 | DASS#15 |  |
| 1. Subjective well-being scale | Factor 1 | SWS#11 | 0.584 | **0.79** |
|  |  | SWS#14 | 0.561 |  |
|  |  | SWS#07 | 0.527 |  |
|  |  | SWS#06 | 0.476 |  |
|  |  | SWS#15 | 0.469 |  |
|  |  | SWS#10 | 0.460 |  |
|  |  | SWS#13 | 0.435 |  |
|  |  | SWS#19 | 0.413 |  |
|  |  | SWS#20 | 0.403 |  |
|  |  | SWS#18 | 0.373 |  |
|  |  | SWS#21 | 0.337 |  |
|  |  | SWS#12 | 0.334 |  |
|  | Factor 2 | SWS#09 | 0.673 | **0.70** |
|  |  | SWS#04 | 0.611 |  |
|  |  | SWS#08 | 0.555 |  |
|  |  | SWS#03 | 0.490 |  |
|  |  | SWS#16 | 0.372 |  |
|  |  | SWS#17 | 0.309 |  |
|  | Factor 3 | SWS#02 | 0.650 | **0.64** |
|  |  | SWS#01 | 0.476 |  |
| 1. Post-traumatic stress disorder symptoms | Factor 1 | PTSD#13 | 0.503 | **0.73** |
|  |  | PTSD#17 | 0.458 |  |
|  |  | PTSD#16 | 0.436 |  |
|  |  | PTSD#10 | 0.429 |  |
|  |  | PTSD#07 | 0.418 |  |
|  |  | PTSD#12 | 0.418 |  |
|  |  | PTSD#15 | 0.409 |  |
|  |  | PTSD#09 | 0.404 |  |
|  |  | PTSD#14 | 0.395 |  |
|  |  | PTSD#11 | 0.392 |  |
|  |  | PTSD#06 | 0.381 |  |
|  |  | PTSD#08 | 0.358 |  |
|  |  | PTSD#05 | 0.340 |  |
| 1. Satisfaction with life scale | Factor 1 | CHS#06 | 0.673 | **0.78** |
|  |  | CHS#07 | 0.625 |  |
|  |  | CHS#05 | 0.613 |  |
|  |  | CHS#04 | 0.580 |  |
|  |  | CHS#08 | 0.552 |  |
|  |  | CHS#03 | 0.519 |  |
|  |  | CHS#02 | 0.445 |  |

3. Reliability of outcome measures

| Latent Variable | Constructs | Item-code | Factor Loading | **Cronbach’s α** |
| --- | --- | --- | --- | --- |
| 1. Menstrual Disorder | Factor 1 | MD#01 | 0.710 | **0.68** |
|  |  | MD#02 | 0.708 |  |
|  |  | MD#03 | 0.596 |  |
|  |  | MD#04 | 0.596 |  |
| 1. Transactional sex practice | Factor 1 | TR_001 | 0.763 | **0.73** |
|  |  | TR_002 | 0.763 |  |
| 1. Premenstrual disorder symptoms | Factor 1 | MS#19 | 0.676 | **0.71** |
|  |  | MS#14 | 0.536 |  |
|  |  | MS#20 | 0.523 |  |
|  |  | MS#15 | 0.515 |  |
|  |  | MS#18 | 0.428 |  |
|  |  | MS#11 | 0.333 |  |
|  |  | MS#02 | 0.593 | **0.75** |
|  |  | MS#03 | 0.577 |  |
|  |  | MS#07 | 0.333 |  |
|  |  | MS#17 | 0.499 | **0.72** |
|  |  | MS#16 | 0.496 |  |
|  |  | MS#22 | 0.449 |  |
|  |  | MS#23 | 0.337 |  |
