# Supplementary material for: Consequences of exposure to sexual harassment among women working in hospitality workplaces in Bahir Dar City, Ethiopia: a structural equation model
Source: Arch Public Health. 2023 Jan 18;81:7. doi: 10.1186/s13690-023-01024-3 (PMC9847057; doi:10.1186/s13690-023-01024-3)
Supplement: Supplementary file 3 — Additional file 3: Supplementary figure 1. [file 13690_2023_1024_MOESM3_ESM.docx]

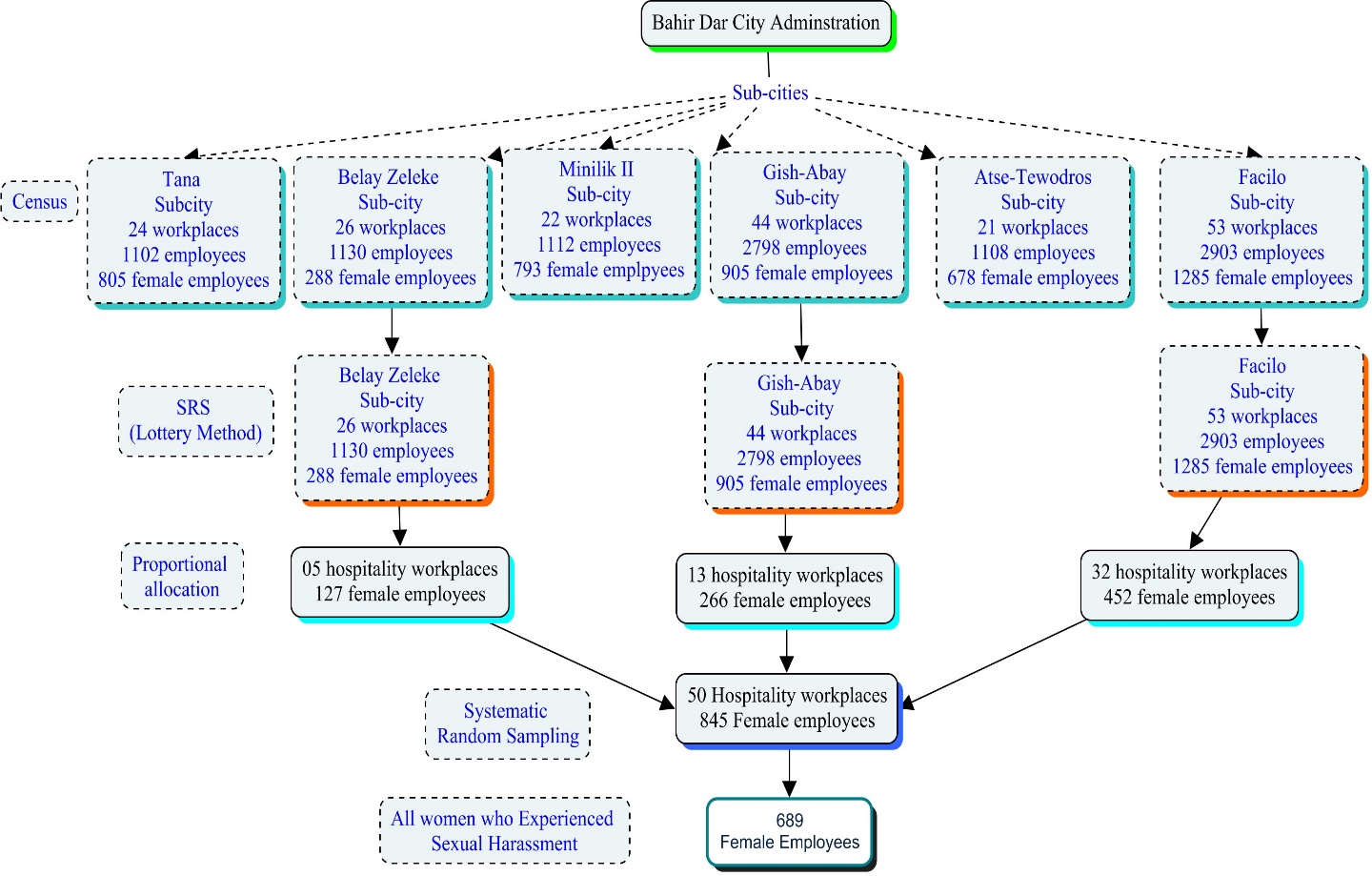


*SRS = Simple Random Sampling*

**Supplementary Figure 1.** Sampling procedure of workplaces and women in Bahir Dar City, Ethiopia, October 1 to December 30, 2021.
